# Production and cross-feeding of nitrite within *Prochlorococcus* populations

**DOI:** 10.1128/mbio.01236-23

**Published:** 2023-07-05

**Authors:** Paul M. Berube, Tyler J. O'Keefe, Anna Rasmussen, Trent LeMaster, Sallie W. Chisholm

**Affiliations:** 1 Department of Civil and Environmental Engineering, Massachusetts Institute of Technology, Cambridge, Massachusetts, USA; 2 Department of Biology, Massachusetts Institute of Technology, Cambridge, Massachusetts, USA; Max Planck Institute for Marine Microbiology, Bremen, Germany

**Keywords:** *Prochlorococcus*, *Synechococcus*, primary nitrite maximum, nitrogen cycle, cross-feeding

## Abstract

*Prochlorococcus* is an abundant photosynthetic bacterium in the open ocean, where nitrogen (N) often limits phytoplankton growth. In the low-light-adapted LLI clade of *Prochlorococcus*, nearly all cells can assimilate nitrite (NO_2_^−^), with a subset capable of assimilating nitrate (NO_3_^−^). LLI cells are maximally abundant near the primary NO_2_^−^ maximum layer, an oceanographic feature that may, in part, be due to incomplete assimilatory NO_3_^−^ reduction and subsequent NO_2_^−^ release by phytoplankton. We hypothesized that some *Prochlorococcus* exhibit incomplete assimilatory NO_3_^−^ reduction and examined NO_2_^−^ accumulation in cultures of three *Prochlorococcus* strains (MIT0915, MIT0917, and SB) and two *Synechococcus* strains (WH8102 and WH7803). Only MIT0917 and SB accumulated external NO_2_^−^ during growth on NO_3_^−^. Approximately 20–30% of the NO_3_^−^ transported into the cell by MIT0917 was released as NO_2_^−^, with the rest assimilated into biomass. We further observed that co-cultures using NO_3_^−^ as the sole N source could be established for MIT0917 and *Prochlorococcus* strain MIT1214 that can assimilate NO_2_^−^ but not NO_3_^−^. In these co-cultures, the NO_2_^−^ released by MIT0917 is efficiently consumed by its partner strain, MIT1214. Our findings highlight the potential for emergent metabolic partnerships that are mediated by the production and consumption of N cycle intermediates within *Prochlorococcus* populations.

**IMPORTANCE:**

Earth’s biogeochemical cycles are substantially driven by microorganisms and their interactions. Given that N often limits marine photosynthesis, we investigated the potential for N cross-feeding within populations of *Prochlorococcus*, the numerically dominant photosynthetic cell in the subtropical open ocean. In laboratory cultures, some *Prochlorococcus* cells release extracellular NO_2_^−^ during growth on NO_3_^−^. In the wild, *Prochlorococcus* populations are composed of multiple functional types, including those that cannot use NO_3_^−^ but can still assimilate NO_2_^−^. We show that metabolic dependencies arise when *Prochlorococcus* strains with complementary NO_2_^−^ production and consumption phenotypes are grown together on NO_3_^−^. These findings demonstrate the potential for emergent metabolic partnerships, possibly modulating ocean nutrient gradients, that are mediated by cross-feeding of N cycle intermediates.

## INTRODUCTION

*Prochlorococcus* and its close relative, *Synechococcus*, are globally abundant non-diazotrophic cyanobacteria that are jointly responsible for approximately 25% of marine net primary production (1). Phytoplankton growth, and thus primary production, is limited by N across much of the surface ocean (2). Given the numerical dominance of *Prochlorococcus*, examining its genotypic and phenotypic diversity in the context of the N cycle can inform our understanding of *Prochlorococcus’* role in marine ecosystems.

*Prochlorococcus* has multiple N assimilation traits, most of which are distributed across cells in *Prochlorococcus* populations. All *Prochlorococcus* appear capable of assimilating ammonium (NH_4_^+^), which is a preferred N source for cyanobacteria likely because it requires less reducing power to assimilate compared to more oxidized N sources (3). Some, but not all, *Prochlorococcus* possess genes enabling the assimilation of urea, cyanate, amino acids, NO_2_^−^, and NO_3_^−^ (4 - 10). It is now evident that there is extensive variability with respect to the N assimilation traits harbored by *Prochlorococcus*. These biological features have the potential to impact N cycling across the vast subtropical ocean gyres—the consequences of which are not well constrained.

The evolutionary history of NO_3_^−^ assimilation in *Prochlorococcus* has deepened our understanding of the selective pressures operating on this organism. Among low-light-adapted *Prochlorococcus*, only the LLI clade has retained the genes for NO_3_^−^ assimilation, while this trait is found in many high-light-adapted clades—e.g., HLI, HLII, and HLVI (10). High-light-adapted cells with NO_3_^−^ assimilation genes appear to be selected for in the surface waters of N-limited systems (9). In contrast, selection for LLI *Prochlorococcus* cells with the capacity to assimilate NO_3_^−^ is not well resolved. This group of *Prochlorococcus* is widely distributed and abundant, often exceeding the combined depth-integrated abundance of other low-light-adapted *Prochlorococcus* (11, 12). Intriguingly, we observed a spatial relationship between LLI cells with the NO_3_^−^ assimilation trait and a peak in NO_2_^−^ concentration in the water column (9).

In stratified marine systems, elevated concentrations of NO_2_^−^ in the mid-euphotic zone are ubiquitous. This feature, the primary NO_2_^−^ maximum layer, is thought to arise from distinct biological processes—either decoupled nitrification (13 - 16) or incomplete NO_3_^−^ reduction by phytoplankton (14, 17). Phytoplankton can be subject to multiple factors affecting the degree to which they excrete NO_2_^−^ during growth on NO_3_^−^ (18). These factors include light (19), growth rate (20), temperature (21), external NO_3_^−^ concentration (22 - 24), Fe limitation (25), and N deficiency (22, 26).

Several features of *Prochlorococcus*’ diversity suggest that NO_2_^−^ cycling could be an important facet of this organism’s ecology, particularly for the abundant low-light-adapted LLI clade of *Prochlorococcus* that dominates the upper reaches of the nitracline. Among these *Prochlorococcus*, the NO_2_^−^ assimilation trait appears to be found in most or all cells while the full pathway for NO_3_^−^ assimilation is only observed in a subset of cells (10). These trait frequencies bear the hallmarks of medium-frequency-dependent selection, which are often governed by cross-feeding interactions (27). Given that LLI *Prochlorococcus* live in the vicinity of the primary NO_2_^−^ maximum layer, we hypothesized that those capable of NO_3_^−^ assimilation have the potential for releasing NO_2_^−^ back into the environment—consistent with what is observed in larger size classes of phytoplankton (18). In this study, we explore incomplete assimilatory NO_3_^−^ reduction by *Prochlorococcus* and further examine the potential for cross-feeding and intra-population cycling of NO_2_^−^, a central intermediate in the N cycle.

## RESULTS AND DISCUSSION

### *Prochlorococcus* strains produce NO_2_^−^ during growth on NO_3_^−^

We first asked whether *Prochlorococcus*, as well as closely related *Synechococcus*, exhibit incomplete assimilatory NO_3_^−^ reduction with concomitant NO_2_^−^ release (18). To address this question, we leveraged a collection of strains (Table S1) with different gene contents (Table S2) and configurations of the NO_3_^−^ and NO_2_^−^ assimilation gene cassette (10). We looked for evidence of extracellular accumulation of NO_2_^−^ during the growth of *Prochlorococcus* and *Synechococcus* strains on NO_3_^−^ in comparison to growth on NH_4_^+^ as the sole N sources. Given that reducing power is ultimately derived from photochemistry in cyanobacteria, we also tested if extracellular accumulation of NO_2_^−^ might be enhanced during growth at lower light intensities—e.g., the reduction of NO_3_^−^ to NO_2_^−^ requires two electrons, while the reduction of NO_2_^−^ to NH_4_^+^ requires six electrons, contributing to a possible bottleneck under light limitation.

**Fig 1:**
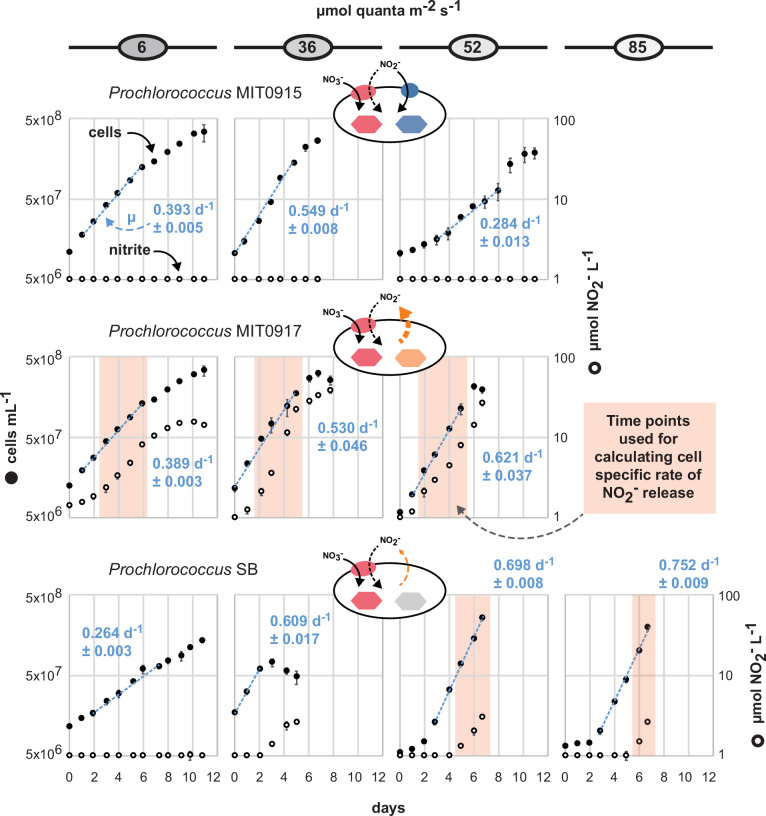
NO_2_^−^ accumulation in triplicate batch cultures of *Prochlorococcus* during growth on NO_3_^−^ as the sole N source over a range of light intensities. Mean cell concentrations are denoted by closed black circles with error bars representing standard deviations. Growth rates (mean and standard deviation of µ for each replicate culture) are shown as blue text with the regression shown as a dashed blue line inclusive of the data points used to calculate growth rates. Mean NO_2_^−^ concentrations are denoted by open circles with error bars representing standard deviations. NO_2_^−^ concentrations below the dynamic range of the assay (<1 µM NO_2_^−^) are plotted on the *x*-axis. Cell-specific rates of NO_2_^−^ release (nmol NO_2_^−^ cell^−1^ d^−1^) were calculated from the log-linear portion of the growth curve (inclusive data points are indicated by red shading).

**Fig 2:**
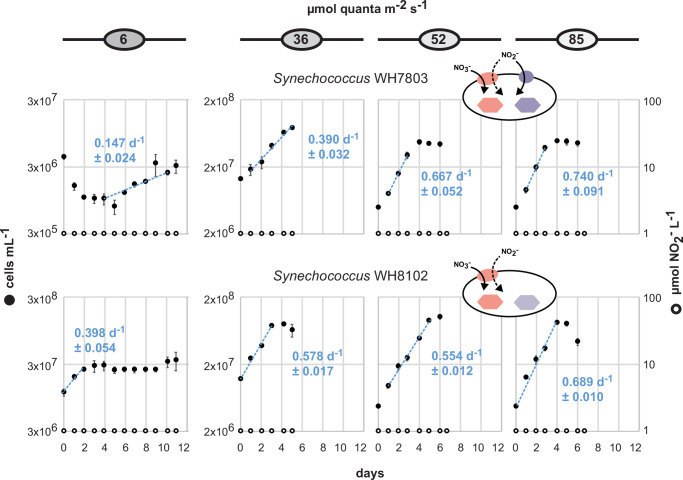
NO_2_^−^ accumulation in triplicate batch cultures of *Synechococcus* during growth on NO_3_^−^ as the sole N source over a range of light intensities. Mean cell concentrations are denoted by closed black circles with error bars representing standard deviations. Growth rates (mean and standard deviation of µ for each replicate culture) are shown as blue text with the regression shown as a dashed blue line inclusive of the data points used to calculate growth rates. Note that the cell concentration range (left *y*-axis) on the plots for cultures grown at 6 µmol photons m^−2^ s^−1^ differs from the plots of cultures grown at higher light intensities. Mean NO_2_^−^ concentrations are denoted by open circles with error bars representing standard deviations. NO_2_^−^ concentrations below the dynamic range of the assay (<1 µM NO_2_^−^) are plotted on the *x*-axis.

For cultures of *Prochlorococcus* MIT0915, we found that NO_2_^−^ concentrations remained below 1 µM (the lower limit of the dynamic range for our assay) at all examined light intensities during growth on NO_3_^−^ (Fig. 1). For the closely related MIT0917 strain, cultures accumulated substantial concentrations of NO_2_^−^ when provided NO_3_^−^ as the sole N source (Fig. 1) compared to growth on NH_4_^+^ (Fig. S1). The high-light-adapted *Prochlorococcus* SB accumulated NO_2_^−^ in the culture medium during growth on NO_3_^−^ but at substantially lower concentrations and with a noticeably greater lag compared to MIT0917 (Fig. 1). In comparison to the *Prochlorococcus* strains examined, neither of the *Synechococcus* strains produced levels of NO_2_^−^ that exceeded 1 µM during growth on NO_3_^−^ or NH_4_^+^ (Fig. 2; Fig. S2).

**Fig 3:**
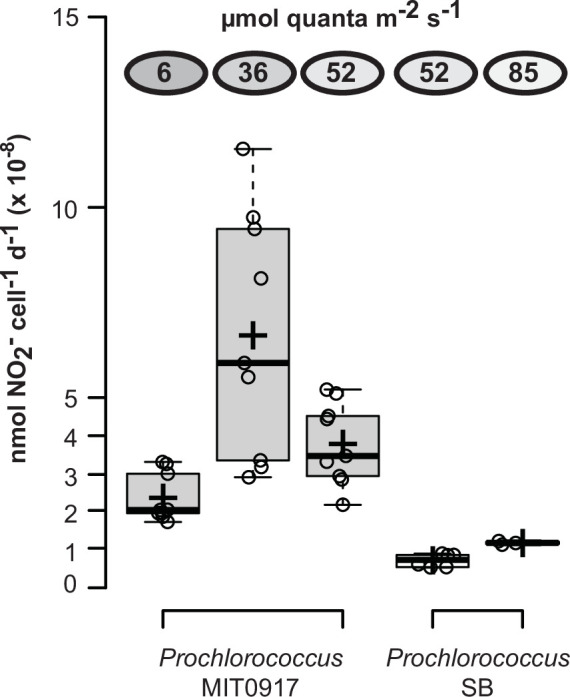
Cell-specific NO_2_^−^ production rates for two strains of *Prochlorococcus*, as a function of light intensity, when grown on NO_3_^−^ as the sole N source. Cell-specific NO_2_^−^ production rates were calculated as the change in NO_2_^−^ concentration relative to the logarithmic mean of cell concentrations—to account for exponential growth—for successive time points during the log-linear portion of the growth curve. Each open circle represents the net cell-specific NO_2_^−^ accumulation rate calculated from a pair of successive time points during exponential growth—inclusive time points are shown in Fig. 1. Means are denoted by crosses, and medians are denoted by solid black lines. The means (± standard deviation) of NO_2_^−^ production rates for MIT0917 were 2.4 × 10^−8^ (±0.64 × 10^−8^), 6.6 × 10^−8^ (±3.2 × 10^−8^), and 3.8 × 10^−8^ (±1.1 × 10^−8^) nmol NO_2_^−^ cell^−1^ d^−1^ at light intensities of 6, 36, and 52 µmol photons m^−2^ s^−1^, respectively. SB produced nitrite at rates of 7.2 × 10^−9^ (±1.7 × 10^−9^) and 1.2 × 10^−8^ (±0.05 × 10^−8^) nmol NO_2_^−^ cell^−1^ d^−1^ at light intensities of 52 and 85 µmol photons m^−2^ s^−1^, respectively. Medians are denoted by solid black lines, and means are denoted by crosses.

We next examined the net cell-specific NO_2_^−^ production rates of MIT0917 and SB during growth on NO_3_^−^. The rates of NO_2_^−^ excretion by the low-light-adapted *Prochlorococcus* MIT0917 were significantly greater than that for the high-light-adapted *Prochlorococcus* SB (Fig. 3). At the same light intensity of 52 µmol photons m^−2^ s^−1^, MIT0917 produced NO_2_^−^ at a fivefold higher rate compared to SB (Fig. 3). The rate of NO_2_^−^ production by MIT0917 was higher at 36 and 52 µmol photons m^−2^ s^−1^ in comparison to the lowest light intensity examined. These rates are necessarily inclusive of potential reuptake of NO_2_^−^ by the NapA transporter encoded by both MIT0917 and SB. NapA has been shown to transport both NO_3_^−^ and NO_2_^−^ in other cyanobacteria (28). Nevertheless, reuptake of NO_2_^−^ could be limited by the fact that the molar equivalents of NO_3_^−^ would exceed that of NO_2_^−^ by at least one order of magnitude throughout the entire growth curve and because of preferential transport of NO_3_^−^ over NO_2_^−^ by NapA as observed for other cyanobacteria (28).

The rates of NO_2_^−^ release we observed for MIT0917 (from 2.4 × 10^−8^ to 6.6 × 10^−8^ nmol NO_2_^−^ cell^−1^ d^−1^) are consistent with rates measured for marine diatoms when normalized to either C quotas (as a proxy for biomass) or cell volumes. In batch culture experiments, *Thalassiosira pseudonana* was observed to produce NO_2_^−^ during growth on NO_3_^−^ at a maximum rate of 2.5 × 10^−14^ µmol s^−1^ cell^−1^ (2.2 × 10^−6^ nmol NO_2_^−^ cell^−1^ d^−1^) (29). Note that diatoms are much larger than cyanobacteria: *T. pseudonana* was observed to have a C quota of 14,000 fg cell^−1^ and a cell volume of 46 µm^−3^ (30), while LLI *Prochlorococcus* were reported to have a C quota of 33 fg cell^−1^ and a cell volume of 0.14 µm^−3^ (31). Thus, *T. pseudonana* cells have approximately 420-fold greater biomass and a 330-fold larger cell volume than LLI *Prochlorococcus* cells. When these ratios in biomass or cell volume are accounted for, MIT0917 produced NO_2_^−^ at normalized rates over threefold higher than *T. pseudonana*.

We then wondered how consequential these rates of NO_2_^−^ release by MIT0917 were with respect to the N requirements of low-light-adapted *Prochlorococcus*. To evaluate this question, we assumed a cellular N quota of 4.3 fg N cell^−1^ for LLI *Prochlorococcus* (31), minimal reuptake of NO_2_^−^, and minimal excretion of other N containing compounds. Based on these assumptions, we estimate that approximately 20–30% of the NO_3_^−^ transported into the cell by MIT0917 during exponential growth may be released as NO_2_^−^, with the balance assimilated into biomass (i.e., the proportion of N as extracellular NO_2_^−^ relative to the combined N in both biomass and extracellular NO_2_^−^). While a broad range of NO_2_^−^ excretion to NO_3_^−^ uptake ratios have been observed previously (18), our estimate for MIT0917 is consistent with values obtained for other phytoplankton (18).

Overall, these data demonstrate that some strains belonging to an abundant low-light-adapted clade of *Prochlorococcus* can release high amounts of NO_2_^−^ when provided NO_3_^−^ as the sole N source. Even high-light-adapted *Prochlorococcus* may release meaningful amounts of NO_2_^−^ when growing on NO_3_^−^. Importantly, the generally positive relationship between light intensity and NO_2_^−^ production rates for both MIT0917 and SB argues against the hypothesis of enhanced NO_2_^−^ release at lower light intensities. To assess alternatives, we looked to the genetic repertoire of these strains as well as the distribution of NO_3_^−^ and NO_2_^−^ assimilating genotypes of LLI *Prochlorococcus* in the wild.

### Contrasting features of NO_3_^−^ and NO_2_^−^ flow in *Prochlorococcus* and *Synechococcus*

**Fig 4:**
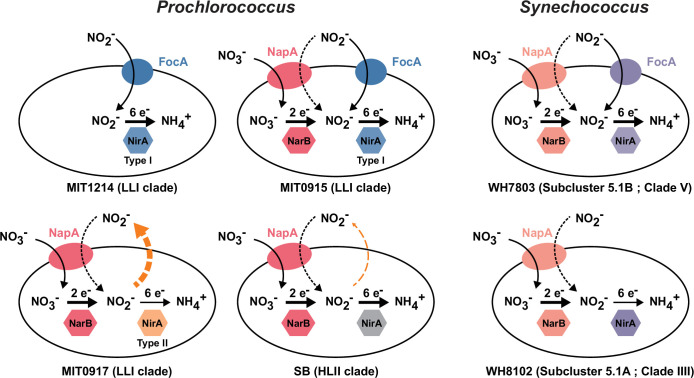
Schematic of cellular inorganic N inputs and outputs for *Prochlorococcus* and *Synechococcus* strains based on physiology data (Fig. 1 and 2) and the genetic inventory of NO_3_^-^ and NO_2_^-^ assimilation genes in their genomes (Table S2). MIT1214 has lost the upstream half of the NO_3_^−^ assimilation pathway but has retained FocA and the type I NirA for the transport and assimilation of NO_2_^−^. MIT0915 has a type I NirA and has also retained the FocA NO_2_^−^ transporter—MIT0915 did not excrete NO_2_^−^ during growth on NO_3_^−^ (Fig. 1). MIT0917 has an alternate version of NirA (type II) compared to other LLI strains and lacks the FocA NO_2_^−^ transporter. SB lacks a dedicated NO_2_^−^ transporter (FocA) but can likely take up NO_2_^−^ using the putatively dual-specific NO_3_^−^/NO_2_^−^ NapA transporter. Both SB and MIT0917 excrete NO_2_^−^ (dashed orange arrow). In comparison to the *Prochlorococcus* strains examined, neither *Synechococcus* strain releases NO_2_^−^ during growth on NO_3_^−^ (Fig. 2). WH7803 possesses the FocA NO_2_^−^ transporter, while WH8102 does not.

Based on what is known about the gene content (Table S2) and physiology (Fig. 1 and 2) of these strains, we can assign hypothetical pathways of inorganic N flow (Fig. 4). For LLI *Prochlorococcus*, these hypothetical pathways map onto the three distinct configurations of the NO_3_^−^ and NO_2_^−^ assimilation gene cassette that we have observed in their genomes (10). Many LLI *Prochlorococcus* have the configuration represented by MIT1214—i.e., they can assimilate both NH_4_^+^ and NO_2_^−^ but not NO_3_^−^. The remaining LLI *Prochlorococcus*—those represented by the NO_3_^−^ assimilating strains, MIT0915 and MIT0917—have contrasting features with regard to NO_2_^−^ production potential (Fig. 4). Under the N-replete conditions we examined, MIT0917 accumulated high extracellular quantities of NO_2_^−^ relative to the proportion of N that was ultimately assimilated into biomass. The closely related MIT0915 strain, however, released negligible quantities of NO_2_^−^ under the same conditions. Besides the absence of a FocA NO_2_^−^ transporter in MIT0917, one key difference between MIT0917 and MIT0915 is the presence of a distinct NirA nitrite reductase in MIT0917 (Fig. 4). Found in other LLI *Prochlorococcus* genomes, this “type II” NirA is phylogenetically divergent from the NirA possessed by MIT1214 and MIT0915 (10). Thus, N transport and reduction kinetics are implicated in the physiological basis for nitrite release by some *Prochlorococcus*.

*Prochlorococcus* SB produced much less NO_2_^−^ during growth on NO_3_^−^ than *Prochlorococcus* MIT0917—and only at the highest light intensities examined—but, similar to MIT0917, SB also lacks the FocA NO_2_^−^ transporter (Fig. 4). *Prochlorococcus* SB belongs to the high-light-adapted HLII clade, which is the most abundant clade of *Prochlorococcus* globally and can represent >90% of depth-integrated *Prochlorococcus* in warm tropical and subtropical waters (12). Although the culturing conditions we employed are quite different than those that cells experience in the wild, HLII clade cells with the potential for NO_2_^−^ release—even if less than that of some LLI clade cells—could have an important impact on NO_2_^−^ cycling in the global ocean due to their sheer abundance. *Synechococcus*, while broadly distributed and responsible for a greater fraction of net primary production compared to *Prochlorococcus*, does not appear to release NO_2_^−^ in batch culture (Fig. 2 and 4). One notable observation is that *Synechococcus* WH8102, like the *Prochlorococcus* strains MIT0917 and SB, lacks the FocA NO_2_^−^ transporter (Table S2), yet unlike the *Prochlorococcus* strains, does not accumulate extracellular NO_2_^−^ (Fig. 2). Given that WH8102 did not release NO_2_^−^, even at the lowest light intensities, we suspect this strain’s phenotype may be imparted by the biochemical features of its NapA transporter and/or NO_3_^−^ and NO_2_^−^ reductases that had evolved under different ecological constraints. Regardless, an important caveat is that the strains we examined represent only a fraction of the diversity of *Synechococcus*.

### Wild populations of LLI clade *Prochlorococcus* comprised coexisting functional types delineated by their use of NO_3_^−^ and NO_2_^−^

In both the subtropical North Pacific and North Atlantic oceans, LLI *Prochlorococcus* (either with or without the capacity for NO_3_^−^ assimilation) often reach maximum abundance within the subsurface chlorophyll maximum layer (12) and in the vicinity of the primary NO_2_^−^ maximum layer (9). Our data now indicate that there is a significant degree of phenotypic diversity with respect to the use of NO_2_^−^ and NO_3_^−^ and that this functional diversity maps onto the genomic diversity of the LLI clade of *Prochlorococcus*. Given these observations, how are LLI *Prochlorococcus* populations structured with respect to the three distinct functional types that we have identified (Fig. 4)? To address this question, we turned to metagenomic data (32) derived from samples collected over 2 years (2003–2004) from the subsurface chlorophyll maximum layer at time-series stations in the North Pacific Subtropical Gyre (Hawai’i Ocean Time-series; HOT) and the North Atlantic Subtropical Gyre (Bermuda Atlantic Time-series Study; BATS). We assessed the frequencies of the *narB, focA*, and both types of the *nirA* gene relative to the *gyrB* gene (a single copy core gene encoding DNA gyrase subunit B) to estimate the fraction of cells in LLI populations that possessed each version of the nitrate assimilation gene cluster (Supplementary Materials and Methods).

**Fig 5:**
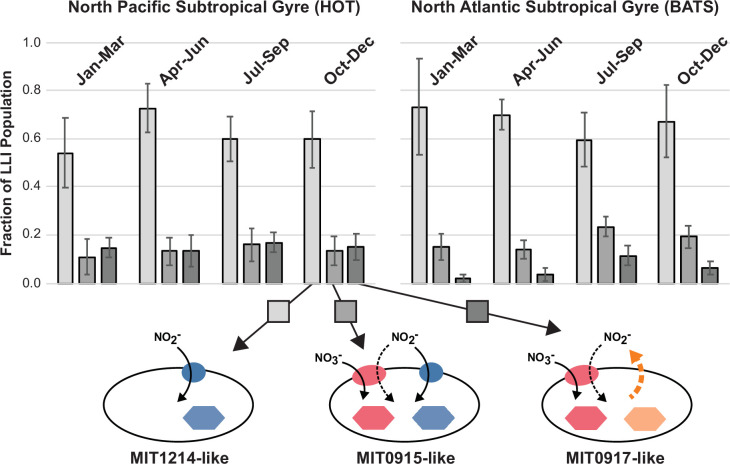
Distribution of functional types in the LLI clade *Prochlorococcus* (based on N flow pathways in Fig. 4) in the North Pacific (HOT) and North Atlantic (BATS) subtropical gyres. Error bars represent the standard deviation of samples grouped by season collected between January 2003 and December 2004. Sample numbers (N) were 6, 6, 4, and 6 at HOT and 5, 5, 4, and 6 at BATS for winter, spring, summer, and autumn, respectively.

At both sites, we observed that MIT1214-like cells (those that assimilate NO_2_^−^ but not NO_3_^−^) dominated the LLI *Prochlorococcus* populations throughout the year and generally exceeded 60% of total LLI genomes (Fig. 5). At HOT in the North Pacific, overall frequencies of each functional type were quite stable on seasonal time scales, with each of the NO_3_^−^ assimilating functional types making up roughly 15% of the population (Fig. 5). In contrast, the seasonal dynamics at BATS in the North Atlantic were readily apparent for the MIT0917-like functional type which waned in the winter and spring and then increased in the summer (Fig. 5). These observations are consistent with previous work that demonstrated higher abundances of HLII *Prochlorococcus* with the potential for NO_3_^−^ assimilation during the summer and autumn in the North Atlantic (9), when overall surface N concentrations were low. The MIT0915-like functional type (the one that did not exhibit incomplete assimilatory NO_3_^−^ reduction in our culture experiments) generally dominated the NO_3_^−^ assimilating functional types in LLI *Prochlorococcus* populations at the North Atlantic’s BATS station (Fig. 5). In contrast, higher frequencies of the MIT0917-like functional type (the one that releases NO_2_^−^) in the North Pacific compared to the North Atlantic suggest that the configuration of the NO_3_^−^ assimilation gene cassette encoding a type II NirA provides a selective advantage to cells in the stable, well-stratified, and generally N-limited waters of the North Pacific Subtropical Gyre.

### *Prochlorococcus* exchange N in co-cultures

Given that LLI *Prochlorococcus* with different N assimilation features (Fig. 4) coexist in the marine environment (Fig. 5), we next assessed whether strains with different N assimilation genotypes could form metabolic dependencies in the laboratory. To achieve this, we co-cultured *Prochlorococcus* MIT1214 (which can use NO_2_^−^ but not NO_3_^−^) with either *Prochlorococcus* MIT0915 or *Prochlorococcus* MIT0917 (each of which can use both NO_3_^−^ and NO_2_^−^) in medium containing NO_3_^−^ as the sole N source.

**Fig 6:**
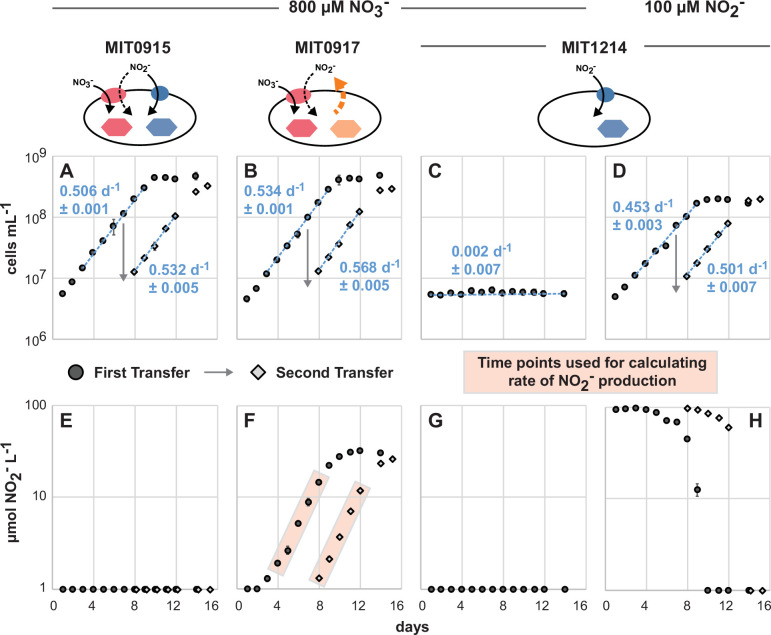
Duplicate pure cultures of MIT0915, MIT0917, and MIT1214 followed over two transfers (transfer 1 in black circles followed by transfer 2 in gray diamonds, with the day of the transfer indicated by the arrow) as contemporaneous controls for co-culture experiments. Mean cell concentrations (**A–D**) for each transfer are shown with error bars representing standard deviations. Growth rates (mean and standard deviation of µ for each replicate culture) are shown as blue text with the regression shown as a dashed blue line inclusive of the data points used to calculate growth rates. Mean NO_2_^−^ concentrations (**E–H**) for each transfer are shown with error bars representing standard deviations. NO_2_^−^ concentrations below the dynamic range of the assay (<1 µM NO_2_^−^) are plotted on the *x*-axis. As pure cultures, MIT0915 and MIT0917 grow using NO_3_^−^ (**A, B**), but only MIT0917 does so with concomitant release of NO_2_^−^ (**E, F**). Red shading denotes data points (**F**) used to calculate cell-specific rates of nitrite release by MIT0917: 4.6 × 10^−8^ (±1.5 × 10^−8^) and 5.4 × 10^−8^ (±0.54 × 10^−8^) nmol NO_2_^−^ cell^−1^ d^−1^ for the first and second transfers, respectively. MIT1214 cannot use NO_3_^−^ as a N source (**C**) but has retained the genes necessary for growth on NO_2_^−^ (**D, H**).

**Fig 7:**
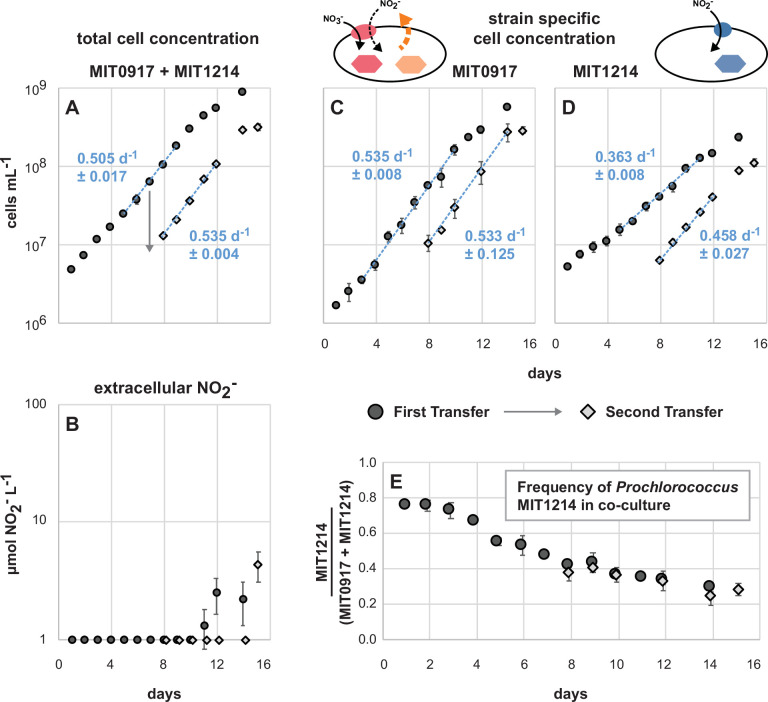
Triplicate co-cultures of *Prochlorococcus* MIT0917 (NO_2_^−^ producer) and *Prochlorococcus* MIT1214 (NO_2_^−^ consumer) over two transfers (transfer 1 in black circles followed by transfer 2 in gray diamonds, with the day of the transfer indicated by the arrow). Each data point represents the mean and standard deviation (error bars) for (**A**) total cell concentration determined by flow cytometry, (**B**) NO_2_^−^ concentration, (**C, D**) strain-specific cell concentration determined by quantitative PCR, and (**E**) the fraction of MIT1214 cells relative to the sum of cell counts assessed by quantitative PCR. Growth rates (mean and standard deviation of µ for each replicate culture) are shown as blue text with the regression shown as a dashed blue line inclusive of the data points used to calculate growth rates. NO_2_^−^ concentrations below the dynamic range of the assay (<1 µM NO_2_^−^) are plotted on the *x*-axis.

Pure cultures of MIT0917 grown at 16 µmol photons m^−2^ s^−1^ of blue light produced nitrite at rates from 4.6 × 10^−8^ to 5.4 × 10^−8^ nmol NO_2_^−^ cell^−1^ d^−1^ (Fig. 6B and F). These rates are between those observed for MIT0917 grown at 6 and 36 µmol photons m^−2^ s^−1^ of white light (Fig. 1 and 3), suggesting that light spectra may have little impact on nitrite production rates. Relative to these pure cultures of MIT0917 (Fig. 6B and F), co-cultures of MIT1214 and MIT0917 did not accumulate NO_2_^−^ in the culture medium during balanced exponential growth (Fig. 7A and B) because any NO_2_^−^ released by MIT0917 was used to fulfill the N requirements of MIT1214. Some NO_2_^−^ accumulation was observed as the MIT1214-MIT0917 co-culture approached stationary phase (Fig. 7A and B), suggesting an imbalance between production and consumption of NO_2_^−^ outside of balanced exponential growth. The growth rates of each of the two strains in co-culture (Fig. 7C and D) were similar to their growth rates in pure culture (Fig. 6B and D), suggesting that MIT0917 could supply nearly all of the N needs of MIT1214. Furthermore, we expect that the growth of MIT1214 under these conditions resembles growth in continuous culture systems—i.e., in co-culture, MIT1214 was likely poised at some degree of N limitation with growth controlled by the rate of NO_2_^−^ release by the partner strain. Although MIT1214 started at a higher cell density than MIT0917, the frequency of the MIT1214 strain settled at roughly 30% of total cell numbers as determined by quantitative PCR (Fig. 7E)—providing additional support for our estimate that MIT0917 partially reduces and excretes up to 30% of the NO_3_^−^ transported into the cell as extracellular NO_2_^−^.

**Fig 8:**
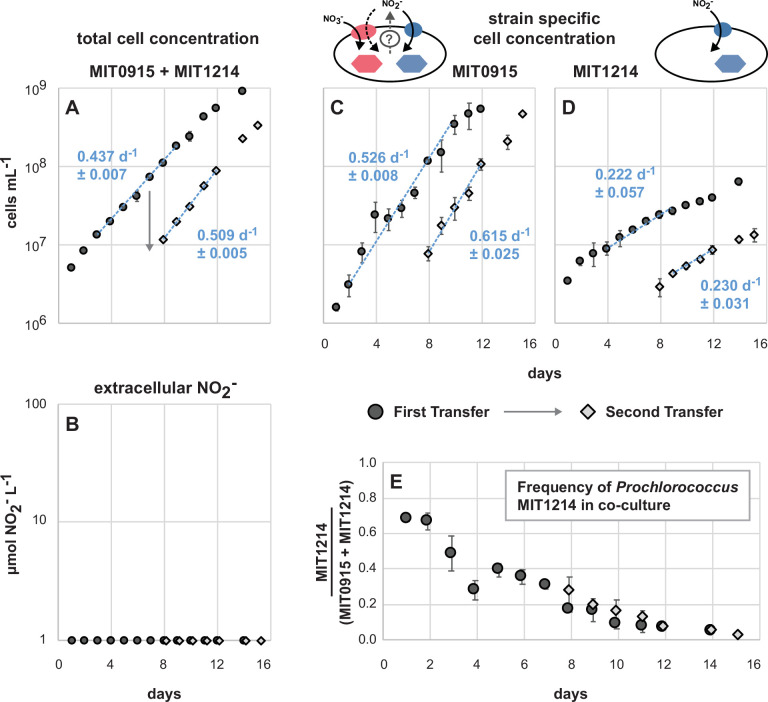
Triplicate co-cultures of *Prochlorococcus* MIT0915 and *Prochlorococcus* MIT1214 over two transfers (transfer 1 in black circles followed by transfer 2 in gray diamonds, with the day of the transfer indicated by the arrow). Each data point represents the mean and standard deviation (error bars) for (**A**) total cell concentration determined by flow cytometry, (**B**) NO_2_^−^ concentration, (**C, D**) strain-specific cell concentration determined by quantitative PCR, and (**E**) the fraction of MIT1214 cells relative to the sum of cell counts assessed by quantitative PCR. Growth rates (mean and standard deviation of µ for each replicate culture) are shown as blue text with the regression shown as a dashed blue line inclusive of the data points used to calculate growth rates. NO_2_^−^ concentrations below the dynamic range of the assay (<1 µM NO_2_^−^) are plotted on the *x*-axis.

When MIT1214 was paired with MIT0915, a strain that does not produce NO_2_^−^ when growing on NO_3_^−^ in pure culture (Fig. 6A and E), we expected the growth of MIT1214 to stop after any carry-over NO_2_^−^ from the inoculum was exhausted. On the contrary, MIT1214 continued to grow (Fig. 8D) but at significantly lower growth rates compared to either growth in the presence of MIT0917 using NO_3_^−^ as the N source (Fig. 7D) or to the growth of MIT1214 in pure culture using NO_2_^−^ (Fig. 6D). As expected, NO_2_^−^ did not accumulate during the growth of the MIT1214-MIT0915 co-culture (Fig. 8A and B). The frequency of the MIT1214 strain was driven to <5% of total cell numbers over the course of two sequential transfers (Fig. 8E), likely as a consequence of the MIT1214 strain’s depressed growth rate in the presence of the MIT0915 strain.

One possible explanation for the MIT1214 strain’s continued slow growth in the presence of MIT0915 may be the release of N-containing organic compounds by its partner strain. *Prochlorococcus*, including those belonging to the LLI clade, have been observed to release some N-containing metabolites (33) that could additionally be packaged into vesicles along with DNA or proteins (34). Alternatively, the continued slow growth of MIT1214 suggests the possibility that MIT0915 does produce low quantities of NO_2_^−^ when growing on NO_3_^−^. Recall that MIT0915 also possesses a FocA NO_2_^−^-specific transporter (Fig. 4)—in contrast to MIT0917—which may facilitate some reuptake of NO_2_^−^ and thus maintain undetectable NO_2_^−^ concentrations in pure culture (Fig. 6A and E). Supply of NO_2_^−^ at rates that keep the concentration of NO_2_^−^ at or below MIT1214’s half-saturation constant (Ks) for growth on NO_2_^−^ (the concentration of NO_2_^−^ at which the growth rate is half the maximum growth rate) would be consistent with its significantly lower growth rate when co-cultured with MIT0915.

### Causes and consequences of incomplete assimilatory NO_3_^−^ reduction by *Prochlorococcus*

Our work has uncovered new potential links between *Prochlorococcus* and the N cycle by demonstrating that some *Prochlorococcus* divert a sizable fraction of transported NO_3_^−^ to extracellular pools of NO_2_^−^ when grown under N-replete batch culture conditions. An intriguing feature of this phenomenon is the degree of phenotypic variability (Fig. 1, 3 and 6F) that maps onto each strain’s particular version of the NO_3_^−^ assimilation gene cluster (10). Given the global abundance of *Prochlorococcus* in the world’s oceans, this new feature of *Prochlorococcus* could have important consequences for our understanding of primary production and N cycle processes that occur in the tropical and subtropical ocean.

While many eukaryotic phytoplankton exhibit incomplete assimilatory NO_3_^−^ reduction, this process is not well constrained for the picocyanobacteria that dominate the expansive subtropical gyres of the open ocean. Why would an organism such as *Prochlorococcus*, which is well adapted to life in oligotrophic habitats, release N back into its environment when this nutrient is often in limited supply? Our observations could be related to laboratory culture conditions where the concentrations of inorganic nutrients are much higher than would be observed in the field. Yet, only one functional type of LLI *Prochlorococcus* (represented by the MIT0917 strain) exhibited incomplete assimilatory NO_3_^−^ reduction. It is possible that the divergent version of the NirA NO_2_^−^ reductase possessed by MIT0917—and similar *Prochlorococcus* in the field—has distinct biochemical features. One hypothesis is that this divergent NirA has a higher substrate affinity, perhaps providing these cells with an advantage under chronically N-limited conditions, such as at HOT in the North Pacific Subtropical Gyre (Fig. 5). Under replete conditions in batch culture, these cells might experience a kinetic bottleneck at the NO_2_^−^ reduction step of the NO_3_^−^ assimilation pathway that results in cellular accumulation of NO_2_^−^ because NO_3_^−^ reduction outpaces the k_cat_ of this divergent NirA. This NO_2_^−^ could then diffuse out of the cell as nitrous acid (HNO_2_) which would make up a small fraction of the intracellular NO_2_^−^ pool at cellular pH (29). Analogous field conditions would be intermittent upwellings of NO_3_^−^ that temporarily increase local substrate supply (35). We assessed the response of MIT0917 to a similar pulse by washing cells with media that was not amended with NO_3_^−^ in order to remove residual N from the cells. These washed cells were starved of NO_3_^−^ for 3 h and then spiked with either 2 µM NO_3_^−^ or 2 µM NH_4_^+^ (Supplementary Materials and Methods). At an NO_3_^−^ concentration that was representative of these intermittent pulses of NO_3_^−^ in the wild, MIT0917 released NO_2_^−^ at a cell-specific rate of 6.1 × 10^−8^ (±0.28 × 10^−8^) nmol NO_2_^−^ cell^−1^ d^−1^ (Fig. S3).

*Prochlorococcus* MIT0917 also lacks the FocA NO_2_^−^-specific transporter that is found in other LLI *Prochlorococcus* (Fig. 4), including the MIT0915 strain that does not exhibit extracellular accumulation of NO_2_^−^ in batch culture. An alternative, but not mutually exclusive, hypothesis for NO_2_^−^ release by MIT0917 is that this strain lacks the capacity for cyclic retention of NO_2_^−^ that may diffuse out of the cell as HNO_2_. It is common for bacteria to employ so-called “futile cycles” to mitigate the loss of nutrients and metabolites across the cell membrane (36, 37). While this process expends energy, it can serve to regulate substrate retention and maintain sufficiently high concentrations of a substrate within the cell. MIT0917 may not require reuptake of NO_2_^−^ if its NirA is optimized for low internal substrate concentrations—in such a scenario, the *focA* gene could have been lost due to a general bias toward gene deletion in the absence of a sufficient selective advantage. The MIT0915 strain, on the other hand, might require a mechanism for cyclic retention of NO_2_^−^ if its NirA has a lower substrate affinity.

Our laboratory-based observations suggest that *Prochlorococcus* may interact with the primary NO_2_^−^ maximum layer in complicated ways. In addition to eroding the primary NO_2_^−^ maximum layer through NO_2_^−^ uptake and assimilation, *Prochlorococcus* also appears to have the potential to amplify the magnitude of the primary NO_2_^−^ maximum layer. At present, it is unclear if *Prochlorococcus* exhibit NO_2_^−^ release in the wild and, if so, what abiotic and biotic factors might influence NO_2_^−^ cycling in these populations. Extrapolating from NO_2_^−^ production rates for both *Prochlorococcus* and ammonia-oxidizing archaea as well as the abundance of marker genes for these microbes in the field, we postulate that NO_2_^−^ production by *Prochlorococcus* could be responsible for some degree of NO_2_^−^ produced in the euphotic zone. *Prochlorococcus* MIT0917 releases NO_2_^−^ at rates from 2 × 10^−8^ nmol NO_2_^−^ cell^−1^ d^−1^ up to 7 × 10^−8^ nmol NO_2_^−^ cell^−1^ d^−1^ (Fig. 3). In comparison, the dominant ammonia-oxidizing microorganism in subtropical open ocean ecosystems, *Candidatus* Nitrosopelagicus brevis (38), produces NO_2_^−^ at a rate of about 2 × 10^−6^ nmol NO_2_^−^ cell^−1^ d^−1^ in batch culture (39). Ammonia-oxidizing archaea are thus expected to produce NO_2_^−^ at 30- to 100-fold higher rates than *Prochlorococcus*, but the latter is often more abundant. For instance, ammonia-oxidizing archaea in the epipelagic zone have been typically observed at abundances of 1,000–6,000 *amoA* gene copies mL^−1^ (16, 40, 41). Low-light-adapted *Prochlorococcus* with the capacity of NO_3_^−^ assimilation, however, can reach abundances of 1,000–40,000 cells mL^−1^ in the vicinity of the subsurface chlorophyll maximum layer (9). Therefore, depending on both rates and cell abundances, *Prochlorococcus* could be responsible from anywhere between <1% to approximately 50% of NO_2_^−^ production in the mid-euphotic zone as a fraction of *Prochlorococcus* and nitrifier-derived NO_2_^-^.

As we have demonstrated, different functional types of *Prochlorococcus* can coexist under conditions where NO_2_^−^ cross-feeding is promoted and NO_2_^−^ accumulation is minimized (Fig. 7). Consequently, NO_2_^−^ production and consumption in *Prochlorococcus* populations might be a cryptic process whereby there is no net accumulation of NO_2_^−^ at steady state. In the wild, *Prochlorococcus* populations could dynamically assemble in response to the availability of N sources of varying redox state as well as in response to community-wide competition for these N sources. Net NO_2_^−^ accumulation might only occur within these populations during periods of perturbation (e.g., changes in light intensity or nutrient supply). Additional study is warranted to examine the conditions under which *Prochlorococcus* populations are either net producers or net consumers of NO_2_^−^ and evaluate how microbial populations and communities modulate the availability of various N sources that ultimately impact production and remineralization processes. At the population level, the dynamic assembly of distinct functional types of *Prochlorococcus* could emerge through interactions that are mediated, in part, by cross-feeding of NO_2_^−^. We posit that trait variability and the selection of complementary functions might facilitate robustness or resiliency in microbial populations. *Prochlorococcus*, as a key primary producer in the tropical and subtropical oceans, offers an extremely valuable lens through which to constrain the rules under which emergent features arise.

## MATERIALS AND METHODS

### Strains

The cultures used in this study included the low-light-adapted *Prochlorococcus* strains MIT0915 (LLI clade), MIT0917 (LLI clade), and MIT1214 (LLI clade), the high-light-adapted *Prochlorococcus* strain SB (HLII clade), as well as *Synechococcus* strains WH8102 (clade III) and WH7803 (clade V) (Table S1).

### NO_2_^−^ production rates

The NO_3_^−^ assimilating strains (MIT0915, MIT0917, SB, WH7803, and WH8102) were grown in triplicate as pure cultures using Pro99 medium (natural seawater base; Sargasso Seawater) with the 800 µM ammonium chloride (NH_4_Cl) omitted and replaced by 800 µM sodium nitrate (NaNO_3_). The cultures were grown in 35 mL of medium in borosilicate glass culture tubes at a temperature of 24°C and under continuous illumination intensities of 6, 36, 52, and 85 µmol photons m^−2^ s^−1^. Given that the strains represent multiple clades of *Prochlorococcus* and *Synechococcus* that span a wide range of depths and light wavelengths in the euphotic zone, we used white light provided by fluorescent lamps. The LLI strains (MIT0915 and MIT0917) had poor and inconsistent growth at the highest light intensity, so these strains were only examined at the three lower light intensities (6, 36, and 52 µmol photons m^−2^ s^−1^). Cultures were monitored daily by removing 0.5 mL of culture in order to determine cell abundances with flow cytometry and NO_2_^−^ concentrations with the Griess colorimetric method (Supplementary Materials and Methods). Net cell-specific NO_2_^−^ production rates were calculated as the change in NO_2_^−^ concentration relative to the logarithmic mean of cell concentrations—to account for exponential growth—for successive time points during the log-linear portion of the growth curve.

### Co-culture experiments

MIT1214 was co-cultured, in triplicate, with either MIT0915 or MIT0917 in 35 mL of medium in borosilicate glass culture tubes using NO_3_^−^ as the sole N source at 24°C. In order to approximate the light intensities and wavelengths that LLI *Prochlorococcus* typically experience in the wild (12, 42, 43), while still maintaining high growth rates, the cultures were grown at 16 µmol photons m^−2^ s^−1^ of continuous blue light (in lieu of white light). As controls, all strains were grown as pure cultures, in duplicate, under the same temperature and light conditions. The MIT1214-MIT0915 and MIT1214-MIT0917 co-cultures, as well as the MIT0915 and MIT0917 pure cultures, used Pro99 medium (natural seawater base; Sargasso Seawater) with 800 µM sodium nitrate (NaNO_3_) as the sole N source. Pure cultures of MIT1214 were grown in Pro99 medium using 100 µM sodium nitrite (NaNO_2_) as the sole N source to serve as a control for growth on NO_2_^−^. Given that some strains of *Prochlorococcus* might be inhibited by NO_2_^−^ (44), a lower concentration of NaNO_2_ was used (instead of 800 µM) to minimize potential growth inhibition due to NO_2_^−^ toxicity. MIT1214 was also grown in Pro99 medium using 800 µM NO_3_^−^ as the sole N source to serve as control for the absence of growth on NO_3_^−^. The co-cultures and pure cultures were sampled daily over two sequential transfers to monitor cell abundances with flow cytometry, NO_2_^−^ concentrations using the Griess method, and to preserve cells on filters for strain-specific cell abundance measurements (Supplementary Materials and Methods). Given that the cell size and fluorescence properties of MIT0915, MIT0917, and MIT1214 overlap when examined using flow cytometry (all are LLI clade cells with similar size and chlorophyll content), we used quantitative PCR to assess the cell abundance of each strain in co-culture. Sample processing, standards, reaction conditions, and amplification efficiencies for the qPCR assays are detailed in the Supplementary Materials and Methods.

## Data Availability

Genome assemblies are available from NCBI GenBank under the following accession numbers: CP114781 (*Prochlorococcus* MIT0915), CP114784 (*Prochlorococcus* MIT0917), CP114777 (*Prochlorococcus* MIT1214), JNAS00000000 (*Prochlorococcus* SB), CT971583 (*Synechococcus* WH7803), BX548020 (*Synechococcus* WH8102). Genome annotations are available from the DOE Joint Genome Institute’s Integrated Microbial Genomes and Microbiomes System (IMG/M) under the following accession numbers: 2681812901 (*Prochlorococcus* MIT0915), 2681812859 (*Prochlorococcus* MIT0917), 2681813567 (*Prochlorococcus* MIT1214), 2606217677 (*Prochlorococcus* SB), 640427149 (*Synechococcus* WH7803) and 637000314 (*Synechococcus* WH8102). Cell density and NO_2_^-^ concentration data are available under dataset 890887 from the Biological and Chemical Oceanography Data Management Office (BCO-DMO).

## SUPPLEMENTAL MATERIAL

Supplemental material mbio.01236-23-s0001.pdf Supplemental Materials and Methods, Tables S1 and S2, and Figures S1 to S3.

ASM does not own the copyrights to Supplemental Material that may be linked to, or accessed through, an article. The authors have granted ASM a non-exclusive, world-wide license to publish the Supplemental Material files. Please contact the corresponding author directly for reuse.
