# Supplementary material for: Production and cross-feeding of nitrite within Prochlorococcus populations
Source: mBio. 2023 Jul 5;14(4):e01236-23. doi: 10.1128/mbio.01236-23 (PMC10470740; doi:10.1128/mbio.01236-23)
Supplement: Supplemental material — s and Methods, Tables S1 and S2, and Figures S1 to S3. [file mbio.01236-23-s0001.pdf]

Supplemental Material

Production and cross-feeding of nitrite within *Prochlorococcus* populations

Paul M. Berube,[a]# Tyler O'Keefe,[a]* Anna Rasmussen,[a]* Trent LeMaster,[a] Sallie W. Chisholm[a,b]

[a]Department of Civil and Environmental Engineering, Massachusetts Institute of Technology, Cambridge, Massachusetts, USA

[b]Department of Biology, Massachusetts Institute of Technology, Cambridge, Massachusetts, USA

#Correspondence to Paul M. Berube, pmberube@mit.edu.

*Present address: Tyler O'Keefe, Department of Earth, Marine, and Environmental Sciences, University of North Carolina at Chapel Hill, Chapel Hill, North Carolina, USA

*Present address: Anna Rasmussen, Department of Earth System Science, Stanford University, Palo Alto, California, USA

This PDF file includes:

- Supplementary Materials and Methods
- Table S1 – *Prochlorococcus* and *Synechococcus* strains
- Table S2 – NCBI and IMG accession numbers and the genetic inventory of nitrate and nitrite assimilation genes for the *Prochlorococcus* and *Synechococcus* strains
- Fig. S1 – Extracellular NO_2^- during the growth of *Prochlorococcus* on NH_4^+
- Fig. S2 – Extracellular NO_2^- during the growth of *Synechococcus* on NH_4^+
- Fig. S3 – NO_2^- release by *Prochlorococcus* MIT0917 at low NO_3^- concentrations
- Supplementary References

SUPPLEMENTARY MATERIALS AND METHODS

Strains. The cultures used in this study are detailed in Table S1. *Prochlorococcus* MIT1214 was isolated from an enrichment culture initiated as part of the Hawaii Ocean Experiment-Dynamics of Light and Nutrients (HOE-DYLAN) VII expedition (UNOLS Cruise ID: KM1217) at coordinates 22.8ºN and -158.0ºE. Unfiltered seawater from 175m was aliquoted into acid-washed 30 mL polycarbonate oakridge tubes and amended with 16 µM sodium nitrate, 1 µM sodium phosphate, 0.117 µM ethylenediaminetetraacetic acid, 0.117 µM iron(III) chloride, 0.0090 µM manganese(II) chloride, 0.0008 µM zinc(II) sulfate, 0.0005 µM cobalt(II) chloride, 0.0003 µM sodium molybdate, 0.0010 µM sodium selenite, and 0.0010 µM nickel(II) chloride. Enrichment cultures were routinely monitored by flow cytometry and those exhibiting growth of a *Prochlorococcus*-like population were transferred into fresh medium and ultimately acclimated to growth on Pro99 medium. As part of our study, MIT1214, MIT0915, and MIT0917 were rendered axenic by dilution to extinction (1). All strains were routinely assayed for heterotrophic contaminants by staining cells with SYBR green and assessing the fluorescence and light scattering properties of both stained and unstained cells using a Guava easyCyte 12HT Flow Cytometer (MilliporeSigma, Burlington, MA, USA) – cultures that did not exhibit the presence of non-photosynthetic cells in the stained samples and had a single cyanobacteria population were presumed axenic and unialgal. All axenic cultures were routinely assessed for purity by confirming a lack of turbidity after inoculation into a panel of purity test broths as described previously (1).

Genome sequencing. The genome of MIT1214 was sequenced as follows. Cells were grown to mid exponential phase and pelleted by centrifugation. DNA was isolated by phenol/chloroform extraction (2). PacBio library preparation and sequencing was carried out by

the MIT BioMicro Center and the UMass Worcester Medical School's Deep Sequencing Core Facility. Assembly of PacBio reads was performed using the hierarchical genome-assembly process (Protocol = RS_HGAP_Assembly.2) as implemented in SMRT Analysis 2.3.0 (3) with the following parameters adjusted: Minimum Polymerase Read Quality = 0.85 and Genome Size = 2000000 bp (default settings were used for all other parameters). Overlapping ends of the assembly were identified using BLAST and the assembly was manually circularized. Circular assemblies were corrected using the RS_Resequencing.1 protocol in SMRT Analysis 2.3.0 (3) with the following parameters: Minimum Polymerase Read Quality = 0.85 and Consensus Algorithm = Quiver. The MIT1214 genome was annotated using IMG Annotation Pipeline version 4 (4, 5), and included in ProPortal CyCOGs 6.0 (6).

Rationale for strain selection. Strains were selected to encompass the observed variations in the NO_3^- and NO_2^- assimilation gene cluster for *Prochlorococcus* and *Synechococcus*. Among strains belonging to the low-light adapted LLI clade of *Prochlorococcus*, we have identified 3 configurations of the NO_3^- and NO_2^- assimilation gene cassette (7), each represented in our study by the MIT1214, MIT0915, and MIT0917 strains (Tables S1 and S2). MIT1214 has the capacity for NO_2^- assimilation, but not NO_3^- assimilation – i.e., it has lost the upstream half of the NO_3^- assimilation pathway, but has retained the downstream half for the assimilation of the more reduced NO_2^-. MIT0915 can assimilate NO_3^- and also possesses a NO_2^- specific transporter (FocA) and a NO_2^- reductase (NirA) that are both closely related to those found in MIT1214. MIT0917 can also assimilate NO_3^-, but in contrast to MIT0915, this strain has a divergent version of NirA and has also lost the gene encoding the FocA NO_2^- transporter (7). We also examined a representative of the high-light adapted HLII clade of *Prochlorococcus*, strain SB (Table S1). SB possesses the full pathway for NO_3^- and

NO_2^- assimilation, except for the FocA NO_2^- transporter (7). The *Synechococcus* strains examined included WH7803 and WH8102, the latter of which is adapted to warm oligotrophic waters and has a range that overlaps with the abundant HLII clade of *Prochlorococcus* (8). Both of these *Synechococcus* strains possess the full pathway for NO_3^- and NO_2^- assimilation, with WH7803 having retained the FocA NO_2^- transporter and WH8102 having lost it.

NO_2^- determination. Extracellular NO_2^- concentrations were determined via the Greiss colorimetric method that reacts sulfanilamide and *N*-(1-naphthyl)ethylenediamine (NED) with NO_2^- to produce a pink-red azo dye with a maximum absorption at a wavelength of 540 nm. A color reagent solution of 1% (10 mg mL^{-1}) sulfanilamide, 5% 12M HCl, and 0.1% (1 mg mL^{-1}) NED was filtered through a 0.2um filter into UV resistant bottles. Aliquots of a 1 mM sodium nitrite ($NaNO_2$) standard solution was stored frozen at -20°C and thawed daily to prepare dilutions spanning 1-50 µM for the generation of a standard curve. To prepare samples for quantification of NO_2^-, 0.15 mL was removed from cultures and filtered through a 96-well 0.45µm MultiScreenHTS HVfilter plate (MilliporeSigma, Burlington, MA, USA) capable of capturing >99% of *Prochlorococcus* cells. Dilutions of the $NaNO_2$ standard were filtered in the same plate as the culture samples to ensure similar treatment. 100uL of filtrate was then transferred from each well to a flat-bottomed, 96-well microplate. An equal volume of color reagent (100 uL) was added to each well, mixed by pipetting, and incubated in the dark for 20 minutes to allow for color development. Absorbance at 540 nm was then determined by using a Synergy 2 Plate Reader (BioTek Instruments, Winooski, VT, USA).

Setup and sampling of co-cultures. Pure cultures of MIT0915, MIT0917, and MIT1214 were passaged twice at 24°C and 16 µmol photons m^{-2} s^{-1} of blue light in Pro99 medium using 800 µM NO_3^- as the sole N source for MIT0915 and MIT0917 and 100 µM NO_2^- as the sole N

source for MIT1214. Cell concentrations were then determined using flow cytometry on a Guava easyCyte 12HT Flow Cytometer (MilliporeSigma, Burlington, MA, USA). Co-cultures were established by inoculating fresh medium with 2 x 10^6 cells mL^{-1} of each strain for a total initial cell concentration of 4 x 10^6 cells mL^{-1}. Pure cultures were inoculated into fresh medium at 4 x 10^6 cells mL^{-1}. Cultures were monitored daily by removing 0.5 mL of culture for flow cytometry and NO_2^- concentration determination as detailed above. Daily samples for quantitative PCR were preserved by filtering 1 mL of culture onto a 25 mm 0.2 µm pore size polycarbonate filter under low vacuum, chasing with 2 mL of qPCR preservation solution (10 mM Tris pH=8, 100 mM EDTA, and 500 mM NaCl), and then transferring the filter to a 2 mL beadbeater tube prior to storage at -80°C. After the initial culture had grown for 7 days, a subsample was transferred into fresh medium at a final cell concentration of 8 x 10^7 cells mL^{-1} (total cells). The initial transfer was monitored for 14 days and the second transfer was monitored for 8 days (i.e., until the cultures began to enter stationary phase as indicated by the daily change in cell concentrations).

Quantitative PCR methods. For MIT0915 and MIT0917, we used an assay that we had previously developed for detection of *narB* in these strains (9). For the detection of MIT1214 we designed a qPCR assay to target the *wckA* gene (encoding a polysaccharide pyruvyl transferase family protein) in MIT1214 that is absent in both MIT0915 and MIT0917. Primers targeting the *wcaK* gene of *Prochlorococcus* MIT1214 were designed using the NCBI Primer-BLAST tool:

Forward Primer, MIT1214_wcaK_283F (5'- GACTACTGCATTTTCGCTGGG - 3')

Reverse Primer, MIT1214_wcaK_402R (5'- ACCTTCAAAACCTCCAACACC - 3')

Samples used to generate standard curves were acquired by growing MIT0915, MIT0917, and MIT1214 to late-exponential phase (approximately 8 x 10^7 cells mL^{-1}), filtering 5 mL of culture

onto a 25 mm 0.2 µm pore size polycarbonate filter under low vacuum, chasing with 3 mL of qPCR preservation solution (10 mM Tris pH=8, 100 mM EDTA, and 500 mM NaCl), and then transferring the filter to a 2 mL beadbeater tube prior to storage at -80°C. Cell concentrations for each culture, at the time of sample filtration, was obtained through flow cytometry. Templates for both experimental cultures and standards were generated by thawing the filters on ice for 2 min, adding 650 µl of 10 mM Tris pH=8, and then beadbeating at 4800 rpm for 2 minutes. Following beadbeating to remove cells from the filter, 500 µl of the buffer was transferred to a 1.5 mL centrifuge tube and heated at 95°C for 15 min to lyse cells. Templates for standard curves were generated by first diluting the resulting template solution to 5.4 x 10^{5} cells $µl^{-1}$ and then performing a serial dilution. All templates were stored at -80°C until use.

The MIT1214 *wcaK* assay was performed in 25 µl reaction volumes with 2.5 µl template and the following final concentrations of reaction components: 12.5 µl QuantiTect SYBR Green PCR Mix (Qiagen, Germantown, Maryland) and 0.5 µmol L^{-1} of each forward and reverse primer. Using a CFX96 Thermocycler (Bio-Rad, Hercules, CA, USA), reactions were pre-incubated at 95°C for 15 min to activate the polymerase and then cycled (40 cycles) at 95°C for 15 s, 57°C for 30 s, and 72°C for 30 s. The MIT0915 and MIT0917 *narB* assays were performed similarly, except for annealing at 60°C for 30 s (9). Amplification efficiencies were 85% for the MIT1214 *wcaK* assay, 90% for the MIT0915 *narB* assay, and 79% for the MIT0917 *narB* assay. Negative controls included MIT0915 and MIT0917 templates for the MIT1214 *wcaK* assay as well as MIT1214 templates for the *narB* assay; no amplification was observed in these negative controls.

Metagenomic derived frequencies of LLI functional types. Paired-end sequencing reads for samples obtained from the subsurface chlorophyll maximum layer at HOT and BATS

(10) were annotated using kaiju 1.7.2 (11) and the MARMICRODB reference database of marine microorganisms (12, 13):

```
kaiju -z 20 \
      -t $DATABASE/nodes.dmp \
      -f $DATABASE/MARMICRODB.fmi \
      -i $READDATADIR/$LIBRARY_1_trimmed.fq \
      -j $READDATADIR/$LIBRARY_2_trimmed.fq \
      -o $LIBRARY_marmicrodb_pairs_kaiju.out -v
```

Reads matching the taxonomic identifier for the LLI clade of *Prochlorococcus* were extracted from the paired-end sequencing reads using seqtk 1.3 (https://github.com/lh3/seqtk). Frequencies of LLI *Prochlorococcus* N assimilation genotypes were determined by further annotation of the taxonomically binned reads using kaiju and the CyCOG v6 database (6). Reads that mapped to the *gyrB*, *narB*, *focA*, type I *nirA*, and type II *nirA* genes were enumerated and normalized to gene length. We assume that each of these genes are found in single copies in *Prochlorococcus* genomes based on their prevalence in the CyCOG v6 database (6). Fractions of LLI *Prochlorococcus* that belonged to each of the 3 functional types (Fig. 4 in the main text) were resolved using the gene length normalized counts of N assimilation marker genes in each metagenome. Specifically, the abundance of MIT1214-like genomes was operationally defined as length-normalized counts of type I *nirA* genes less the length-normalized counts of *narB* genes. MIT0917-like genomes were defined as the length-normalized counts of type II *nirA* genes. MIT0915-like genomes were defined as the length-normalized counts of *narB* genes less the length-normalized counts of type II *nirA* genes. Each of these values was divided by the length-normalized counts of the *gyrB* gene, a single copy core gene in LLI *Prochlorococcus*, in order to obtain the fraction of the LLI *Prochlorococcus* population represented by each functional type. Seasonality of the frequency of each functional type in LLI *Prochlorococcus*

populations was assessed by binning these data into 4 subsets based on sample collection month – winter (January through March), spring (April through June), summer (July through September), and autumn (October through December).

NO_2^- production at low NO_3^- concentrations. The cell-specific NO_2^- production rate of *Prochlorococcus* MIT0917 was further assessed in cultures amended with 2 µM NO_3^- to explore the potential occurrence of incomplete assimilatory NO_3^- reduction at environmentally relevant NO_3^- concentrations. Growth medium was prepared by using autoclaved seawater obtained from surface waters of the N-limited South Pacific Subtropical Gyre during the MV1015 cruise. Inorganic N in the surface mixed layer at the time of seawater collection was undetectable (14). The sterile seawater was amended with 160 µM sodium nitrate, 10 µM sodium phosphate, and 0.2x of Pro99 trace metals (15). *Prochlorococcus* MIT0917 was grow in duplicate in 250 mL of this medium in 500 mL polyethylene bottles until the cells reached late exponential phase. The cultures were then pelleted at 10,000 RPM in a JA-14 rotor for 15 minutes at 22°C. The spent medium was decanted, leaving approximately 2 mL of residual liquid. The cells were then resuspended in 100 mL of medium which lacked a NO_3^- amendment and pelleted again in order to remove residual NO_3^- from the biomass. The cells were washed a second time in this very low NO_3^- medium and then the cells were resuspended in 50 mL of the very low NO_3^- medium. Assuming that 2 mL of residual liquid remained after decanting following each centrifugation step, these washing steps would result in decreasing the residual amended NO_3^- in the culture to < 0.003 µM.

Cell concentrations for the resuspended MIT0917 cells were determined by preparing 3 independent dilutions of each culture and counting cells using a Guava easyCyte 12HT Flow Cytometer (MilliporeSigma, Burlington, MA, USA). The washed cells were starved of N for 3

hours prior to initiation of the N pulse. Each culture was diluted to a final concentration of 1 x 10^{-8} cells mL^{-1} in duplicate 50 mL of very low NO_3^- medium. One of these duplicate cultures was spiked with 2 µM of sodium nitrate (to assess NO_2^- production at an environmentally relevant NO_3^- concentration) and the other duplicate culture was spiked with 2 µM of ammonium chloride (to serve as a control). Every 12 minutes over a time course of 2 hours, 4.5 mL of culture was removed from each tube and filtered through a 0.2 µm PES syringe filter into a 15 mL tube and then frozen at -20°C.

Within 24 hours, the samples were thawed and NO_2^- concentrations assessed using an AA3 HR continuous segmented flow analyzer (SEAL Analytical, Mequon, WI, USA), fitted with a 520 nm bandpass filter, by employing the G-384-08 method (SEAL Analytical) for the determination of NO_3^- and NO_2^- in water and seawater. The system wash was composed of artificial seawater (481 mM NaCl, 28 mM $MgSO_4$, 27 mM $MgCl_2$, 10 mM $CaCl_2$, and 9 mM KCl prepared using 18 megohm water). The color reagent, which reacts with NO_2^- to produce a pink-red azo dye, was composed of 1% sulfanilamide, 0.05% *N*-(1-naphthyl)ethylenediamine (NED), and 10% phosphoric acid prepared using 18 megohm water. Based on 15 independent measurements of zero calibrator samples and low concentration standards, the limit of blank (LOB; defined as the mean of blank samples plus 1.645 times the standard deviation of blank samples) of our assay was 9 nM NO_2^- and the limit of detection (LOD; defined as the LOB plus 1.645 times the standard deviation of a 15 nM NO_2^- standard) of our assay was 12 nM NO_2^-. The limit of quantitation of our assay was 30 nM NO_2^- (LOQ; defined as the lowest concentration of our standards that yields <10% coefficient of variation).

Table S1. *Prochlorococcus* and *Synechococcus* strains used in this study.

Strain	Genus	Subcluster/Clade	Inorganic N Assimilation	Original Reference	Rendered Axenic
MIT0915	*Prochlorococcus*	Clade LLI	NH_4^+, NO_2^-, NO_3^-	(9)	This Study
MIT0917	*Prochlorococcus*	Clade LLI	NH_4^+, NO_2^-, NO_3^-	(9)	This Study
MIT1214*	*Prochlorococcus*	Clade LLI	NH_4^+, NO_2^-	This Study	This Study
SB	*Prochlorococcus*	Clade HLII	NH_4^+, NO_2^-, NO_3^-	(16)	(1)
WH7803	*Synechococcus*	Subcluster 5.1B; Clade V	NH_4^+, NO_2^-, NO_3^-	(17)	(17)
WH8102	*Synechococcus*	Subcluster 5.1A; Clade III	NH_4^+, NO_2^-, NO_3^-	(17)	(17)

* A physiology study by Hawco et al. (18) was the first published instance of MIT1214, but as the original isolators of this strain, herein we report the methods and metadata for the isolation and genome sequencing of MIT1214.

Table S2. Inventory of nitrate and nitrite assimilation genes based on genomic data (6, 7) for the *Prochlorococcus* and *Synechococcus* strains used in this study.

	MIT0915	MIT0917	MIT1214	SB	WH7803	WH8102
**Nitrite Transport and Reduction**						
focA	●		●		●	
nirA	●	●	●	●	●	●
**Nitrate Transport and Reduction**						
napA	●	●		●	●	●
narB	●	●		●	●	●
**Molybdopterin Cofactor Biosynthesis**						
moaA	●	●		●	●	●
moaC	●	●		●	●	●
moaB	●	●		●	●	●
mobA	●	●		●	●	●
moeA	●	●		●	●	●
moaE	●	●		●	●	●
moaD	●	●		●	●	●
**Genomic and Annotation Data**						
**NCBI GenBank Accession**	CP114781	CP114784	CP114777	JNAS00000000	CT971583	BX548020
**IMG Genome ID**	2681812901	2681812859	2681813567	2606217677	640427149	637000314

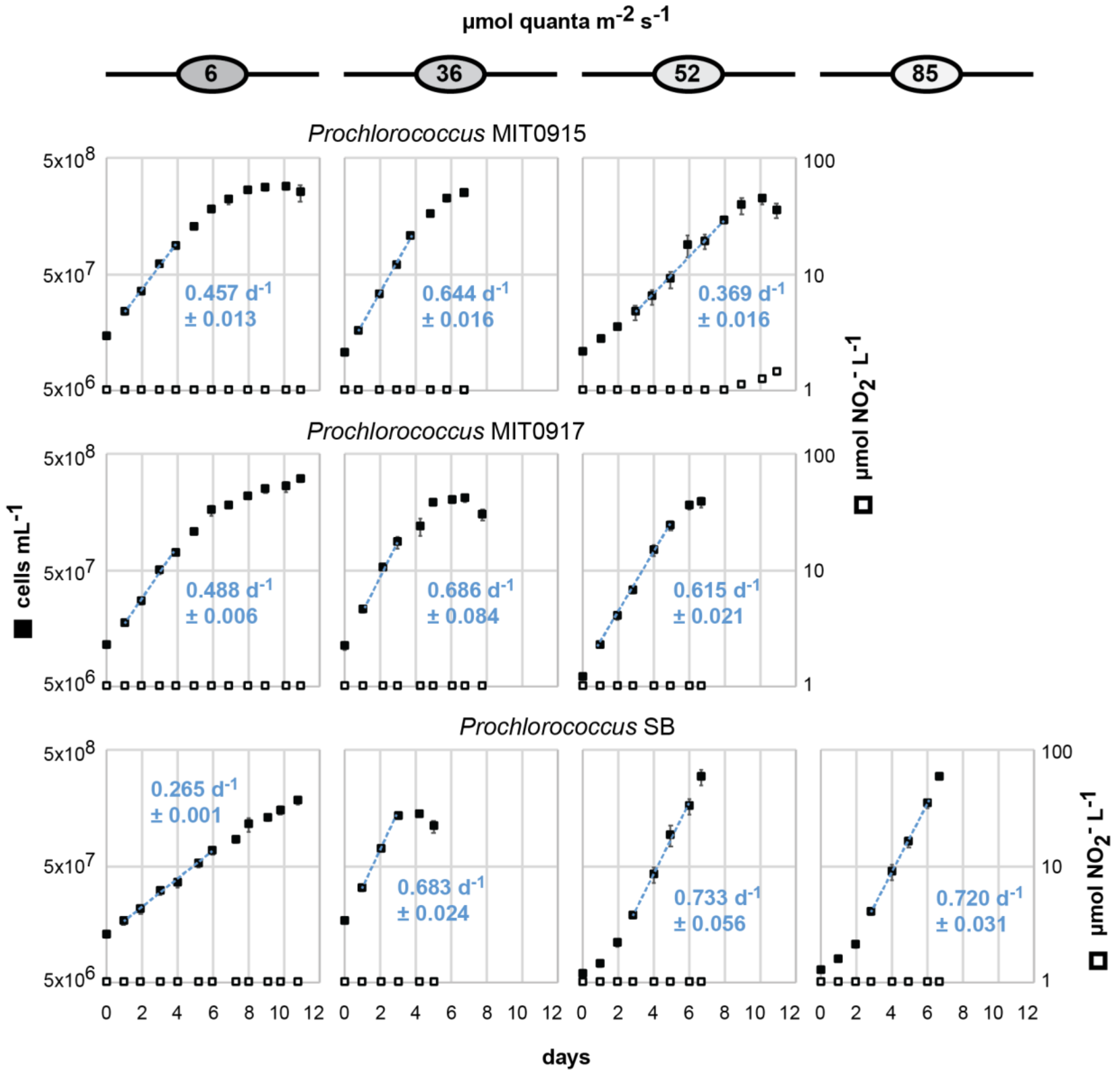

Fig. S1. Growth rates and extracellular NO_2^- concentrations for triplicate batch cultures of *Prochlorococcus* grown on NH_4^+ as the sole N source over a range of light intensities. Mean cell concentrations are denoted by closed black squares with error bars representing standard deviations. Growth rates (mean and standard deviation of µ for each replicate culture) are shown as blue text with the regression shown as a dashed blue line inclusive of the data points used to calculate growth rates. Mean NO_2^- concentrations are denoted by open squares with error bars representing standard deviations. NO_2^- concentrations below the dynamic range of the assay (< 1 µM NO_2^-) are plotted on the x-axis.

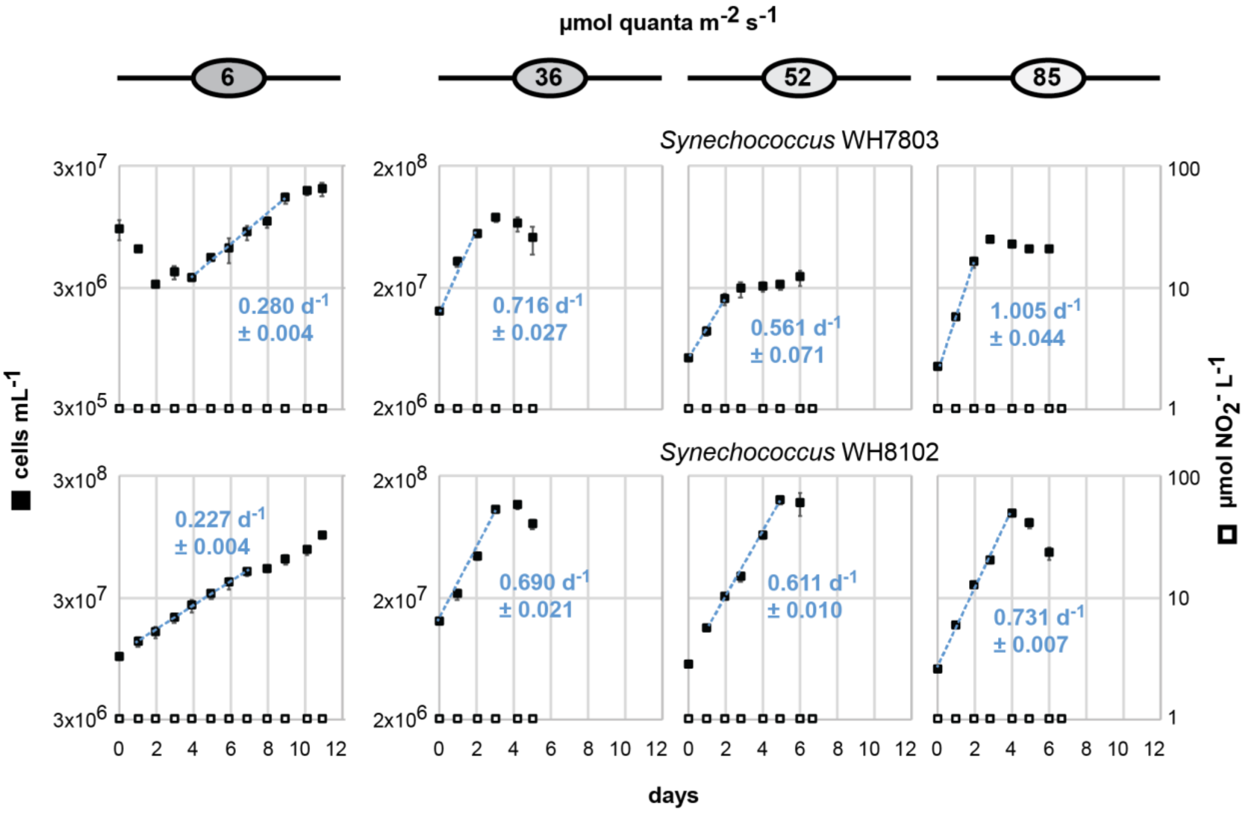

Fig. S2. Growth rates and extracellular NO_2^- concentrations for triplicate batch cultures of *Synechococcus* grown on NH_4^+ as the sole N source over a range of light intensities. Mean cell concentrations are denoted by closed black squares with error bars representing standard deviations. Growth rates (mean and standard deviation of µ for each replicate culture) are shown as blue text with the regression shown as a dashed blue line inclusive of the data points used to calculate growth rates. Note that the cell concentration range (left y-axis) on the plots for cultures grown at 6 µmol photons m^{-2} s^{-1} differs from the plots of cultures grown at higher light intensities. Mean NO_2^- concentrations are denoted by open squares with error bars representing standard deviations. NO_2^- concentrations below the dynamic range of the assay (< 1 µM NO_2^-) are plotted on the x-axis.

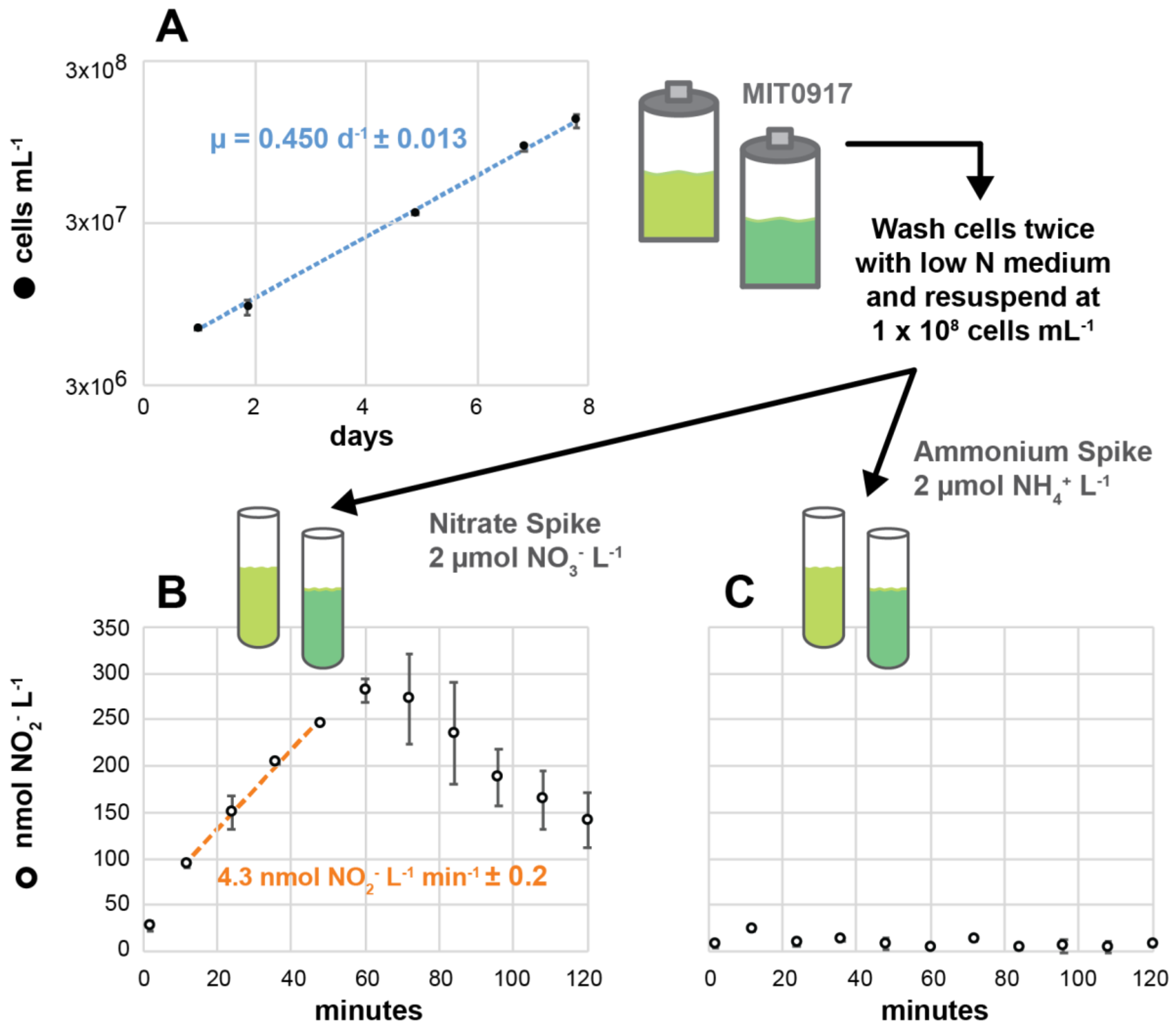

Fig. S3. NO_2^- production by *Prochlorococcus* MIT0917 in the presence of 2 µM NO_3^-. Mean cell concentrations for duplicate 250 mL cultures (A) are denoted by closed black circles with error bars representing standard deviations. Growth rate (mean and standard deviation of µ for each replicate culture) is shown as blue text with the regression shown as a dashed blue line inclusive of the data points used to calculate growth rates. Following the washing and resuspension of cells in low NO_3^- medium, NO_2^- concentrations were determined for duplicate cultures that were spiked with either 2 µM NO_3^- (B) or 2 µM NH_4^+ (C). NO_2^- concentrations are denoted by open black circles with error bars representing standard deviations. The bulk rate of NO_2^- accumulation (mean and standard deviation of the slope for each replicate culture spiked with 2 µM NO_3^-) is shown as orange text with the regression shown as a dashed orange line inclusive of the data points used to calculate the slope (B). The cell-specific NO_2^- production rate (accounting for a cell concentration of 1 x 10^8 cells mL^{-1}) was 6.1 x 10^{-8} (+/- 0.28 x 10^{-8}) nmol NO_2^- $cell^{-1}$ d^{-1}.

SUPPLEMENTARY REFERENCES

1. Berube PM, Biller SJ, Kent AG, Berta-Thompson JW, Roggensack SE, Roache-Johnson KH, Ackerman M, Moore LR, Meisel JD, Sher D, Thompson LR, Campbell L, Martiny AC, Chisholm SW. 2015. Physiology and evolution of nitrate acquisition in *Prochlorococcus*. ISME J 9:1195–1207.

2. Wilson K. 2001. Preparation of genomic DNA from bacteria. Curr Protoc Mol Biol 56:2.4.1–2. 4.2. https://doi.org/10.1002/0471142727.mb0204s56

3. Chin C-S, Alexander DH, Marks P, Klammer AA, Drake J, Heiner C, Clum A, Copeland A, Huddleston J, Eichler EE, Turner SW, Korlach J. 2013. Nonhybrid, finished microbial genome assemblies from long-read SMRT sequencing data. Nat Methods 10:563–569.

4. Markowitz VM, Chen I-MA, Palaniappan K, Chu K, Szeto E, Pillay M, Ratner A, Huang J, Woyke T, Huntemann M, Anderson I, Billis K, Varghese N, Mavromatis K, Pati A, Ivanova NN, Kyrpides NC. 2014. IMG 4 version of the integrated microbial genomes comparative analysis system. Nucleic Acids Res 42:D560–7.

5. Chen I-MA, Markowitz VM, Chu K, Palaniappan K, Szeto E, Pillay M, Ratner A, Huang J, Andersen E, Huntemann M, Varghese N, Hadjithomas M, Tennessen K, Nielsen T, Ivanova NN, Kyrpides NC. 2017. IMG/M: integrated genome and metagenome comparative data analysis system. Nucleic Acids Res 45:D507–D516.

6. Berube PM, Biller SJ, Hackl T, Hogle SL, Satinsky BM, Becker JW, Braakman R, Collins SB, Kelly L, Berta-Thompson J, Coe A, Bergauer K, Bouman HA, Browning TJ, De Corte D, Hassler C, Hulata Y, Jacquot JE, Maas EW, Reinthaler T, Sintes E, Yokokawa T, Lindell D, Stepanauskas R, Chisholm SW. 2018. Single cell genomes of *Prochlorococcus,*

Synechococcus, and sympatric microbes from diverse marine environments. Sci Data 5:180154.

7. Berube PM, Rasmussen A, Braakman R, Stepanauskas R, Chisholm SW. 2019. Emergence of trait variability through the lens of nitrogen assimilation in *Prochlorococcus*. eLife 8:e41043.

8. Kent AG, Baer SE, Mouginot C, Huang JS, Larkin AA, Lomas MW, Martiny AC. 2019. Parallel phylogeography of *Prochlorococcus* and *Synechococcus*. ISME J 13:430–441.

9. Berube PM, Coe A, Roggensack SE, Chisholm SW. 2016. Temporal dynamics of *Prochlorococcus* cells with the potential for nitrate assimilation in the subtropical Atlantic and Pacific oceans. Limnol Oceanogr 61:482–495.

10. Biller SJ, Berube PM, Dooley K, Williams M, Satinsky BM, Hackl T, Hogle SL, Coe A, Bergauer K, Bouman HA, Browning TJ, De Corte D, Hassler C, Hulston D, Jacquot JE, Maas EW, Reinthaler T, Sintes E, Yokokawa T, Chisholm SW. 2018. Marine microbial metagenomes sampled across space and time. Sci Data 5:180176.

11. Menzel P, Ng KL, Krogh A. 2016. Fast and sensitive taxonomic classification for metagenomics with Kaiju. Nat Commun 7:11257.

12. Becker JW, Hogle SL, Rosendo K, Chisholm SW. 2019. Co-culture and biogeography of *Prochlorococcus* and SAR11. ISME J 13:1506–1519.

13. Hogle SL. 2019. MARMICRODB database for taxonomic classification of (marine) metagenomes (Version 1.0.0) [Data set]. Zenodo https://doi.org/10.5281/zenodo.3520509

14. Letelier R. 2011. Nutrients from R/V Melville MV1015 in the South Pacific from Arica, Chile to Easter Island. from November to December 2010 (C-MORE project). Biological

and Chemical Oceanography Data Management Office (BCO-DMO). (Version 04 May 2011) Version Date 2011-05-04. http://lod.bco-dmo.org/id/dataset/3472

15. Moore LR, Coe A, Zinser ER, Saito MA, Sullivan MB, Lindell D, Frois-Moniz K, Waterbury J, Chisholm SW. 2007. Culturing the marine cyanobacterium *Prochlorococcus*. Limnol Oceanogr Meth 5:353–362.

16. Shimada A, Nishijima M, Maruyama T. 1995. Seasonal appearance of *Prochlorococcus* in Suruga Bay, Japan. J Oceanogr 51:289–300.

17. Waterbury JB, Watson SW, Valois FW, Franks DG. 1986. Biological and ecological characterization of the marine unicellular cyanobacterium *Synechococcus*, p 71–120. *In* Platt T, Li WKW (ed), Photosynthetic Picoplankton, Department of Fisheries and Oceans, Ottawa.

18. Hawco NJ, Fu F, Yang N, Hutchins DA, John SG. 2021. Independent iron and light limitation in a low-light-adapted *Prochlorococcus* from the deep chlorophyll maximum. ISME J 15:359–362.
